# Initial training in cold snare polypectomy for residents using a new training model

**DOI:** 10.1055/a-2134-9823

**Published:** 2023-08-21

**Authors:** Hiroki Kato, Makoto Kobayashi, Motoyoshi Yano

**Affiliations:** Department of Gastroenterology, Yokkaichi Municipal Hospital, Mie, Japan

We have previously reported that the use of the *E* ndoscopist & *A* ssistant's *S* imulator dr *Y* lab (EASY; Tanac Co. Ltd., Gifu, Japan) ( Fig. 1 ) was useful in recruiting residents 1 . The version of EASY described in this report (EASY CSP) is an endoscopic simulator for resection by snare and clip closure developed by Matsuzaki and Tsunemi ( Fig. 2 ). The procedure can be practiced on the device, which has been developed for training in polypectomy and suturing techniques. It allows for easy training with simple set up.

**Fig. 1:**
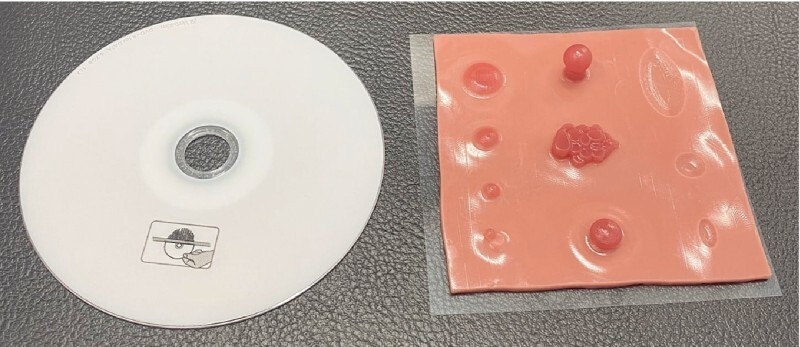
Photograph of the EASY sheet, which is the same size as a compact disc.

**Fig. 2:**
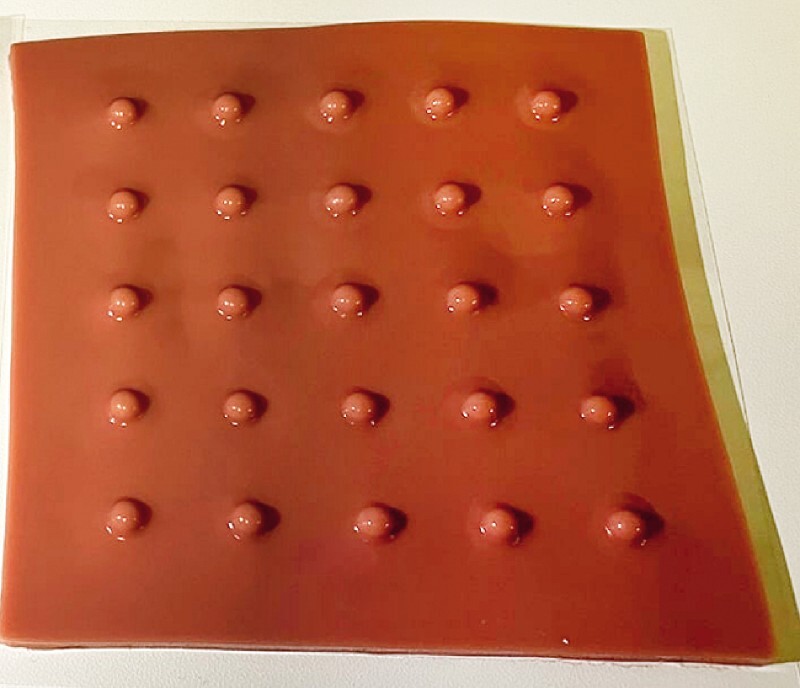
Photograph of the new EASY CSP sheet that was used this time to simulate cold snare polypectomy (CSP).

EASY was used for cold snare polypectomy (CSP) by two of our residents who had no colonoscopy experience ( Video 1 ). On the 25 polyp models, the first one in each row was used for instruction, and the remaining four polyps in the row were then used for practice. This was repeated for five rows. The time required to perform CSP on each of the 25 polyp models was measured. We found that the resection time gradually decreased and, through the use of EASY, a reduction in procedure time was obtained.

**Video 1** This video shows how residents can use the EASY CSP sheet for initial training in cold snare polypectomy (CSP).


Adequate instruction for the 25 CSPs resulted in an increase in procedural proficiency. The use of the EASY CSP sheet before performing actual CSP on a patient is likely to be useful from a medical safety perspective. It is easy to introduce, inexpensive, and may become a standard part of training endoscopists in the future. In addition to the standard endoscopy model, adding guidance by EASY appears to be useful as a part of endoscopist education.

Endoscopy_UCTN_Code_TTT_1AU_2AB

## Notes

Endoscopy E-Videos

https://eref.thieme.de/e-videos
*E-Videos* is an open access online section of the journal *Endoscopy* , reporting on interesting cases and new techniques in gastroenterological endoscopy. All papers include a high-quality video and are published with a Creative Commons CC-BY license. Endoscopy E-Videos qualify for HINARI discounts and waivers and eligibility is automatically checked during the submission process. We grant 100% waivers to articles whose corresponding authors are based in Group A countries and 50% waivers to those who are based in Group B countries as classified by Research4Life (see: https://www.research4life.org/access/eligibility/ ). This section has its own submission website at https://mc.manuscriptcentral.com/e-videos
